# Prospects for high gain inertial fusion energy: an introduction to the first special edition

**DOI:** 10.1098/rsta.2020.0006

**Published:** 2020-10-12

**Authors:** P. A. Norreys, C. Ridgers, K. Lancaster, M. Koepke, G. Tynan

**Affiliations:** 1 Atomic and Laser Physics sub-Department, Department of Physics, University of Oxford, Clarendon Laboratory, Parks Road, Oxford OX1 3PU, UK; 2 Central Laser Facility, UKRI-STFC Rutherford Appleton Laboratory, Harwell Campus, Didcot, Oxfordshire OX11 0QX, UK; 3 Department of Physics, University of York, Heslington, York YO10 5DD, UK; 4 Department of Physics and Astronomy, White Hall Box 5315, West Virginia University, Morgantown, WV 26506-6315, USA; 5 Department of Mechanical and Aerospace Engineering, University of California San Diego, 9500 Gilman Dr., La Jolla, CA 92093, USA

**Keywords:** inertial confinement fusion, inertial fusion energy, fast ignition, central hot spot, direct drive, indirect drive

## Abstract

A European consortium of 15 laboratories across nine nations have worked together under the EUROFusion Enabling Research grants for the past decade with three principle objectives. These are: (a) investigating obstacles to ignition on megaJoule-class laser facilities; (b) investigating novel alternative approaches to ignition, including basic studies for fast ignition (both electron and ion-driven), auxiliary heating, shock ignition etc.; and (c) developing technologies that will be required in the future for a fusion reactor. The Hooke discussion meeting in March 2020 provided an opportunity to reflect on the progress made in inertial confinement fusion research world-wide to date. This first edition of two special issues seeks to identify paths forward to achieve high fusion energy gain.

This article is part of a discussion meeting issue ‘Prospects for high gain inertial fusion energy (part 1)’.

## 1. Introduction

Inertial fusion energy requires the 1000-fold compression of matter to ultra-high densities and temperatures to mimic the compressional effect of gravity in the sun, nature’s very effective nuclear fusion reactor. By irradiating and imploding a small spherical shell containing isotopes of hydrogen (deuterium and tritium) in the laboratory, either directly using intense nanosecond-duration ultra-violet laser beams or indirectly by immersing the shell in an intense bath of re-radiated longer-wavelength X-rays, the shell compresses almost instantaneously. At maximum compression, the fuel’s own inertia, e.g. the tendency of matter to resist sudden acceleration, permits enough delay between implosion and subsequent explosion for the strong but short-range nuclear force to dominate and fuse a sufficient fraction of the isotope pairs into a helium nucleus.

During each isotope-pair fusion event, a sudden and intense release of energy occurs because the rest mass of the two fusion products (a helium nucleus and an energetic neutron) is less than the combined masses of the two fusion ingredients (a deuterium ion and a tritium ion). (Remember that the gained energy equals the mass difference multiplied by the square of the speed of light, i.e. *E* = *mc*^2^.) The higher the rate of fusion reactions, the greater the energy generated beyond that expended to drive the compression in the first place. This heat, if captured in a surrounding blanket, as in a conventional power plant, can drive a steam turbine that generates electric power.

All approaches to nuclear fusion must satisfy a criterion, memorized by fusion scientists, which states that the product of the density, temperature and confinement time of the fusion fuel must exceed a predictable threshold value for the energy gain to be positive instead of negative. To maintain the fuel’s required ultra-high density and temperature, all fusion devices must inject energy into the fuel and minimize the degree to which the fusion fuel comes into thermal contact with the surrounding reactor vessel.

Magnetic-fusion confinement relies on strong electromagnets controlling charged-particle orbits over ultra-long time-scales at conditions of intermediate energy density. Such research is now underway at the Culham Centre for Fusion Energy with experiments at the Joint European Torus (JET), the spherical tokamak MAST and StEP programmes at Culham and will soon be underway in experiments at the International Thermonuclear Experimental Reactor (ITER) in France. Research underway at the Lawrence Livermore National Laboratory’s National Ignition Facility in the USA, and at the Laser MegaJoule facility in France, uses inertial confinement over ultra-short time-scales at conditions of high energy density.

Inertial fusion relies on high-frequency repetition energy injection. An applied burn-wave impacts and propagates through the fuel shell. The burn-wave thermalizes the kinetic energy of the imploding fuel shell by the time the burn-wave is forced to stagnate at the shell centre. Commercializing inertial fusion energy requires highly reproducible injection of a fusion shell to a precise target position and the application of 5–10 burn-wave repetitions per second.

The scientific and technological progress in exploratory inertial confinement fusion research has been impressive, relative to its modest government-provided research budget, over the past two decades. Priority has been given to the assembly and understanding of the high-energy-density conditions in the compressed fuel. The enabling technologies required for inertial fusion energy applications has received second priority. These technologies include high-repetition-rate lasers, heavy-ion beam drivers, pulsed-power magnetic-compression generators, and high-reproducibility cryogenic-target ‘assembly and qualification’.

The Hooke Discussion meeting, held in early March 2020 at the Royal Society in London, brought together about eighty of the world’s leading scientists and policy advocates to review the state of the art in inertial confinement fusion research and to begin preparations for the next generation facilities that advance the goal of high fusion energy gain, i.e. high rate of fusion reactions and high compression of individual fuel shells.

The discussion meeting itself divided into four sessions: potential benefits, progress, current status and commercialization. A number of thought-provoking articles are presented here, in this first of two editions of the Philosophical Transactions of the Royal Society A, that illuminate the scope of the debate in the meeting’s sessions.

Steven Rose *et al.*’s article ‘Modelling burning thermonuclear plasmas’ [1] illustrates the enormous scientific promise of investing in inertial fusion research from a fundamental physics perspective, irrespective of whether inertial fusion can become a commercially competitive source for electricity generation in future. Similarly, Andrew Randewich *et al.*’s article ‘Inertial confinement fusion—a defence context’ [2] describes the requirements for nuclear stockpile stewardship and the relationship with forefront studies in this field of research.

Then, in the scholarly article ‘How might controlled fusion fit into the emerging low-carbon energy system of the mid-21st century?’ [3], George Tynan *et al.* review the current status of electricity demand in the United States and conclude that smaller modular nuclear fusion devices are more likely to fit into existing electricity markets. Nicholas Hawker in his article ‘A simplified economic model for inertial fusion’ [4] presents results from a 14-variable simulation tool. He argues that high gain devices are required in order for inertial fusion to become commercially competitive. Andrew Holland in his article ‘Political and commercial prospects for Inertial Fusion Energy’ [5] summarizes the interest in the commercial development opportunities by industrial companies, with the welcome establishment of the Fusion Industry Association as an independent advocacy voice. Stephen Dean brings his wealth of experience in his lifelong support of fusion energy research in Washington DC to bear in his article ‘Beyond the physics and demonstration of fusion ignition’ [6].

Vladimir Tikhonchuk *et al.* in their article ‘Progress and opportunities for the Inertial Fusion Energy in Europe’ [7] provides a very useful summary of existing research activities within the European Union, mainly based upon microphysics studies related to the direct drive approach. The special issue then goes on to include a number of specific research articles where current-day microphysics obstacles to ignition are discussed in more detail. These include the role of the electrothermal instability for magnetic field generation in the central hot-spot of the fusion target in the article ‘Magnetic field generation from composition gradients in inertial confinement fusion fuel’ [8] by James Sadler *et al.* Similarly, the electrothermal instability is also discussed in the ablation-front seeding of Rayleigh–Taylor instabilities in the direct-drive approach in ‘The importance of laser pulse-ablator interaction dynamics prior to ablation plasma phase in ICF studies’ [9] article by E. Kaselouris and others in Michael Tatarakis’ research group. The work on ‘Crossed beam energy transfer between optically smoothed laser beams in inhomogeneous plasmas’ [10] by Stefan Hueller *et al.* discusses the physics of this source of energy transfer between laser beams in plasma.

The article ‘Reflectivity and spectral shift from laser plasmas generated by high-contrast, high-intensity KrF laser pulses’ [11] by Istvan Foldes *et al.* presents some of the first energy transport studies using high contrast ratio ultra-violet laser pulses. It is likely that high energy ultra-violet petawatt class lasers will be required in future for the fast ignition approach to inertial fusion. Jie Zhang *et al.*’s article ‘Double-cone ignition scheme for inertial confinement fusion’ [12] presents a new initiative that is now funded and whose construction is underway in the People’s Republic of China, also based upon the fast ignition inertial fusion approach.

The first edition of this special issue concludes with Stephen Obenschain *et al.*’s very interesting article ‘Direct-drive with the Argon Fluoride laser as a path to high-gain with sub-megajoule laser energy’ [13] describing the potential benefits of using still shorter wavelengths (193 nm) that are available with argon-fluoride excimer lasers as the driver to achieve high gains for inertial fusion energy.

## Data accessibility

This article has no additional data.
